# Prolonged tenofovir treatment of macaques infected with K65R reverse transcriptase mutants of SIV results in the development of antiviral immune responses that control virus replication after drug withdrawal

**DOI:** 10.1186/1742-4690-9-57

**Published:** 2012-07-17

**Authors:** Koen K A Van Rompay, Kristin A Trott, Kartika Jayashankar, Yongzhi Geng, Celia C LaBranche, Jeffrey A Johnson, Gary Landucci, Jonathan Lipscomb, Ross P Tarara, Don R Canfield, Walid Heneine, Donald N Forthal, David Montefiori, Kristina Abel

**Affiliations:** 1 California National Primate Research Center, University of California, Davis, CA, 95616, USA; 2 Department of Surgery, Duke University, Durham, NC, 27710, USA; 3 Division of HIV/AIDS Prevention, National Center for HIV, STD and Tuberculosis Prevention, Centers for Disease control and Prevention, Atlanta, GE, 30333, USA; 4 Division of Infectious Diseases, Department of Medicine, University of California, Irvine School of Medicine, Irvine, CA, 92697, USA; 5 Department of Microbiology and Immunology, School of Medicine, University of North Carolina, Chapel Hill, NC, 27599, USA

**Keywords:** Tenofovir, PMPA, SIV, Functional cure, Antiretroviral, HIV

## Abstract

**Background:**

We reported previously that while prolonged tenofovir monotherapy of macaques infected with virulent simian immunodeficiency virus (SIV) resulted invariably in the emergence of viral mutants with reduced *in vitro* drug susceptibility and a K65R mutation in reverse transcriptase, some animals controlled virus replication for years. Transient CD8+ cell depletion or short-term tenofovir interruption within 1 to 5 years of treatment demonstrated that a combination of CD8+ cell-mediated immune responses and continued tenofovir therapy was required for sustained suppression of viremia. We report here follow-up data on 5 such animals that received tenofovir for 8 to 14 years.

**Results:**

Although one animal had a gradual increase in viremia from 3 years onwards, the other 4 tenofovir-treated animals maintained undetectable viremia with occasional viral blips (≤ 300 RNA copies/ml plasma). When tenofovir was withdrawn after 8 to 10 years from three animals with undetectable viremia, the pattern of occasional episodes of low viremia (≤ 3600 RNA/ml plasma) continued throughout the 10-month follow-up period. These animals had low virus levels in lymphoid tissues, and evidence of multiple SIV-specific immune responses.

**Conclusion:**

Under certain conditions (i.e., prolonged antiviral therapy initiated early after infection; viral mutants with reduced drug susceptibility) a virus-host balance characterized by strong immunologic control of virus replication can be achieved. Although further research is needed to translate these findings into clinical applications, these observations provide hope for a functional cure of HIV infection via immunotherapeutic strategies that boost antiviral immunity and reduce the need for continuous antiretroviral therapy.

## Background

Considering the bleak prognosis for HIV-infected patients during the early years of the epidemic, our current ability to manage HIV infection with antiretroviral therapy (ART) and other supportive care represents a triumph of research and modern medicine [1]. Despite the ongoing discovery of compounds with better efficacy, safety and dosage regimens, long-term ART is still complicated by issues such as cost, compliance and drug resistance, which are often more pronounced in resource-limited settings where treatment options are limited.

Accordingly, the quest continues to find strategies that would reduce or totally eliminate the need for antiretroviral drugs. The observation that HIV-1 infection seems to have been cured in the so-called Berlin patient by irradiation (to treat leukemia) followed by a bone marrow transplant from a donor with the Δ32 CCR5 mutation provided important proof-of-concept and has initiated a “race for the cure” to develop drug-based strategies to purge out viral reservoirs and ultimately cure HIV infection, i.e. a sterilizing cure [2-4].

The ultimate goal of a practical, cheap and safe method to completely cure HIV infection, however, is likely to be years away. Therefore, an alternative goal is a functional cure, in which virus is not eliminated but is controlled sufficiently by antiviral immune responses so that drug treatment can be withdrawn for prolonged periods of time [4]. Because withdrawal of ART generally leads to a rapid viral rebound, a variety of interventions have been explored to boost immunological control prior to ART withdrawal; these strategies include structured treatment interruptions, active immunization and immune reconstitution strategies [5-8]. While many of these strategies showed some modest efficacy, the benefits were often variable or transient, or the strategy was technically challenging. Accordingly, none of the immunotherapeutic strategies is yet ready to be implemented beyond the confinement of closely monitored clinical trials.

Simian immunodeficiency virus (SIV) infection of macaques is a well-established animal model of HIV infection. It has proved useful to gain a better understanding of antiretroviral therapy and viral reservoirs, and to explore the efficacy and durability of immunotherapeutic strategies to help the immune system in controlling virus replication (reviewed in [9]).

In a series of studies, we demonstrated previously that prolonged tenofovir monotherapy of macaques infected with virulent SIVmac251 or RT-SHIV resulted invariably in the emergence of viral mutants with a K65R mutation in reverse transcriptase [10-12]. Nonetheless, a significant proportion of animals were able to eventually suppress plasma viremia to low or undetectable levels for years, with increasing difficulty to isolate virus *in vitro* from lymphocytes obtained from blood or lymphoid tissues. Such control of viremia was never observed in untreated animals infected with these wild-type or K65R viruses [10,11,13-17]. Transient CD8+ cell depletion or short-term tenofovir interruption within 1 to 5 years of the onset of treatment demonstrated that a combination of potent CD8+ cell-mediated immune responses and continued tenofovir therapy was required for this sustained suppression of viremia [10,11,13,15].

Here, we report follow-up data on a cohort of five such animals that received tenofovir therapy for 8 to 14 years; this long duration of antiretroviral therapy is, to our knowledge, unprecedented in the SIV macaque model. We report that although one animal gradually lost control on K65R virus replication while still on tenofovir therapy, the other animals resembled long-term non-progressors because their immune system continued to control virus replication even after withdrawal of tenofovir therapy.

## Results

### Historical overview of animals

**Figure 1:**
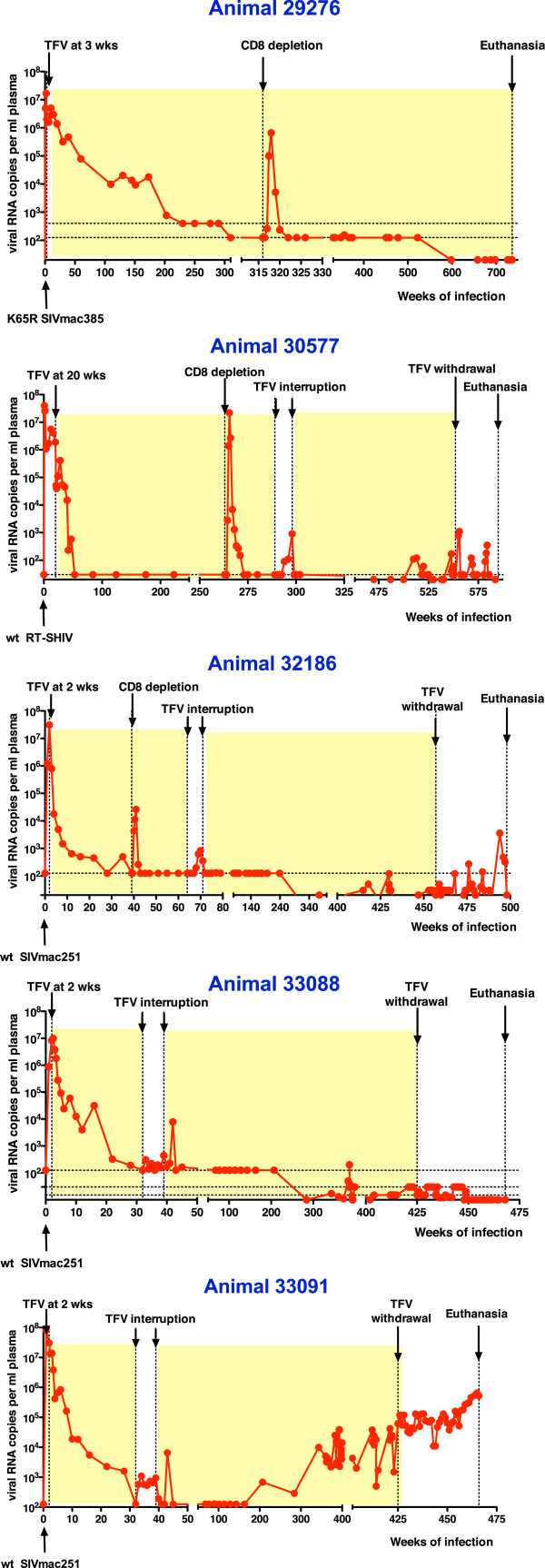
**Plasma viral RNA levels in infected macaques before and during tenofovir treatment, and following tenofovir withdrawal.** Animals were infected with wild-type (wt) or K65R RT viral mutants as described in Table 1. The shaded areas represent periods of tenofovir treatment. Horizontal dotted lines represent the consecutively lower detection limits of the viral RNA assays (500, 125, 30, 10 copies/ml) during the historical course of the experiments.

As described in detail previously [10,11,13-15] and summarized in Table 1 and Figure 1, five animals were infected at birth or at juvenile age with either wild-type SIVmac251 (n = 3), a K65R isolate derived from SIVmac251 (n = 1) or RT-SHIV (a chimeric SIV containing HIV-1 RT). Subsequently, the animals were started on prolonged tenofovir treatment, and all had been able to reach undetectable plasma viremia. All animals had previously been depleted transiently of CD8+ cells by administration of the anti-CD8 antibody cM-T807, either early or late during the course of tenofovir therapy. Four of the five animals had also received a short treatment interruption that had resulted in an increase in viremia, which became undetectable again when tenofovir therapy was reinstated.

**Table 1:** Summary of history of tenofovir-treated SIV-infected macaques

**Animal number**	**Virus inoculum**^**a**^	**Age of virus infection**	**Start of tenofovir**^**b, c**^	**CD8+ cell depletion**^**d**^	**Temporary tenofovir interruption**	**Permanent tenofovir withdrawal**	**Time of euthanasia**
29276	SIVmac-K65R	3 days	3 wks	316 wks	None	None	736 wks
30577	RT-SHIV	19 months	20 wks	263 wks	289 to 298 wks	552 wks	595 wks
32186	SIVmac251	17 months	2 wks	39 wks	64 to 71 wks	457 wks	498 wks
33088	SIVmac251	12 months	2 wks	2 wks	32 to 39 wks	425 wks	468 wks
33091	SIVmac251	12 months	2 wks	2 wks	32 to 39 wks	425 wks	466 wks

^**a**^ Animals were inoculated orally or intravenously with wild-type SIVmac251 or RT-SHIV, with the exception of animal 29276, which was inoculated with a K65R SIV isolate (SIVmac385) derived from tenofovir-treated SIVmac251-infected animals, as described previously [10,11,13]. All animals had reached undetectable viremia (with sometimes transient blips). With exception of animal 33091, which eventually had a gradual increase in viremia, all other animals maintained undetectable or low viremia even after tenofovir withdrawal.

^b^ Tenofovir was initially given to all these animals at a once-daily regimen of 30 mg/kg body weight, administered subcutaneously, with subsequent dosage reductions to stable maintenance regimens, as described earlier [10,13,15].

^c^ Times are expressed as weeks after SIV inoculation.

^d^ Transient CD8+ cell depletion was performed by administration of the monoclonal antibody cM-T807 as described earlier [11].

### MHC class I typing

Animals were typed for MHC class I alleles that in some studies correlated with lower viremia (Mamu-A*01, -B*08, -B*17) or higher viremia (Mamu-B*01) of some, but not all, SIV isolates, particularly SIVmac239 [18-21]. Their impact on control of other SIV isolates, such as the SIVmac251 stocks used in the current study is not clear [10]. In this study, we observed no consistent pattern of protective alleles (Table 2). Two animals with good virological control (numbers 29276 and 32186) had none of the protective alleles; in contrast, the only animal with two of the alleles (33091) was the one that gradually lost control of virus replication (see further).

**Table 2:** Typing of MHC class I alleles of SIV-infected animals

**Animal #**	**A01**	**A02**	**A08**	**A11**	**B01**	**B03**	**B04**	**B08**	**B17**	**MHC score**^**a**^
29276	**-**	**-**	**-**	**-**	**-**	**-**	**-**	**-**	**-**	**0**
32186	**-**	**-**	**-**	**-**	**-**	**-**	**-**	**-**	**-**	**0**
30577	**+**	**-**	**-**	**+**	**-**	**-**	**-**	**-**	**-**	**+1**
33088	**-**	**-**	**-**	**-**	**+**	**-**	**-**	**+**	**-**	**0**
33091	**+**	**-**	**-**	**-**	**-**	**-**	**-**	**+**	**-**	**+2**
CNPRC frequency (%)^b^	18.8	13.8	13.0	9.1	29.9	1.2	1.5	7.1	11.2	

^a^The MHC score was calculated by adding one point for each protective allele (Mamu-A*01, B*08, B*17) and subtracting a point for Mamu-B*01, which has been associated with higher viral levels; other alleles are given a zero score [18-21].

^b^Frequency of alleles in the general rhesus macaque colony of the California National Primate Research Center; there was no statistically significant difference in frequency between the 5 SIV-infected animals and the general frequency for any of these alleles (p values > 0.5).

### Virological & clinical outcome

For consistency, we used a working definition of viral blip quite similar to the one used commonly in humans [22], but taking into account that we monitored animals more frequently (sometimes more than once per week) than humans: a viral blip was defined as a viral RNA load measurement of 50–1000 copies/mL lasting less than 3 weeks, that was preceded and followed by another HIV RNA load measurement of <50 copies/mL.

Animal 29276 maintained undetectable viremia (< detection limits of 10–15 RNA copies per ml plasma) from week 322 onwards, with no detection of viral blips (Figure 1; Table 3). This animal had chronic renal problems, triggered by a prolonged high-dose tenofovir regimen during the first years of life, when the relationship between drug levels and renal toxicity was still poorly understood. Although the renal insufficiency was largely managed by dosage reduction, it still progressed slowly, as described previously [14,15]. However, at approximately 14 years of age (736 weeks of infection), while still on tenofovir therapy, an adverse reaction to another medication aggravated the renal toxicity and the animal had to be euthanized. Tissues collected at time of euthanasia showed low virus levels that were undetectable or at the limit of detection (Table 4).

**Table 3:** Analysis of viral blips in animals with low viremia during tenofovir treatment and after tenofovir withdrawal

	**During tenofovir treatment**				**After tenofovir withdrawal**				
**Animal number**^**a**^	**Time window (weeks of infection)**^**b**^	**Number of time points for plasma viral RNA analysis**	**Number of time points with plasma viral RNA ≥ 50 copies per ml**	**Peak plasma virus copies per ml (time point)**	**Time window (weeks of infection)**^**b**^	**Number of time points for plasma viral RNA analysis**	**Number of time points with plasma viral RNA ≥ 50 copies per ml**	**Peak plasma virus copies per ml (time point)**	**One-sided*p*value for frequency of viremia ≥ 50 copies/ml during*versus*after tenofovir treatment**
29276^c^	598-736	16	0	NA^c^	NA^c^	NA^c^	NA^c^	NA^c^	NA^c^
30577	299-552	39	12	270 (513 wks)	553-595 wks	40	7	1,100 (556 wks)	*p* = 0.13
32186	316-457	39	4	300 (418 wks)	458 -498 wks	40	9	3,600 (494 wks)	*p* = 0.13
33088	284-425	38	2	200 (386 wks)	426-468 wks	40	0	30 (435 wks)	*p* = 0.23

**Table 4:** Virus levels in blood and tissues at time of euthanasia

**Animal number**	**Plasma**	**PBMC**		**Spleen**		**Ax LN**		**Ing LN**		**Mes LN**		**Thymus**		**Tonsil**		**Jejunum**		**Colon**	
	**RNA**	**DNA**	**RNA**	**DNA**	**RNA**	**DNA**	**RNA**	**DNA**	**RNA**	**DNA**	**RNA**	**DNA**	**RNA**	**DNA**	**RNA**	**DNA**	**RNA**	**DNA**	**RNA**
29276	<15	4	<2	2	1	3	2	3	<2	<1	<1	NA	NA	NA	NA	NA	NA	NA	NA
30577	< 10	7	<2	<3	<3	4	<1	4	<1	7	<2	<2	<2	3	<1	<2	<2	2	<2
32186	20	15	<1	5	<1	40	9	7	90	30	200	<70	160	300	13,500	60	7	10	50
33088	< 10	6	<1	2	<1	8	10	4	1	5	3	<2	<2	3	1	<2	<2	<4	<4
33091	530,000	280	470	370	5,700	320	13,000	340	11,000	520	10,000	<2	50	570	30,000	60	640	50	160

Cell-free levels in plasma are expressed as RNA copies per ml plasma. Cell-associated levels are expressed as number of viral RNA and DNA copies per 100,000 cell equivalents (determined by total copy number of CCR5 divided by two). < indicates below limit of detection (based on the detection limit of 30 viral RNA or DNA copies per reaction and the cell equivalents for the particular sample).

Ax LN = axillary lymph node, Ing LN = inguineal lymph node; Mes LN = mesenteric lymph node; NA = not available.

During tenofovir treatment, another 3 animals (30577, 32186 and 33088) continued to have mostly undetectable plasma viremia (below the cut-off of 10–30 copies per ml), with occasional viral blips between 50 and 200 RNA copies/ml (Figure 1, Table 3). Testing of PBMC for cell-associated virus showed that most samples had detectable but low viral DNA levels, with occasional detection of low SIV RNA levels (Table 5).

**Table 5:** Summary of plasma and PBMC-associated virus levels shortly before and after tenofovir withdrawal

	**30577**			**32186**			**33088**			**33091**		
	**Plasma**	**PBMC**		**Plasma**	**PBMC**		**Plasma**	**PBMC**		**Plasma**	**PBMC**	
**Time after tenofovir withdrawal**	**RNA**	**DNA**	**RNA**	**RNA**	**DNA**	**RNA**	**RNA**	**DNA**	**RNA**	**RNA**	**DNA**	**RNA**
- 24 wks	< 10	4	1	< 10	1	< 1	< 10	4	4	4,300	20	5
- 12 wks	< 15	7	2	< 10	< 1	< 1	< 15	3	< 1	38,000	20	20
- 4 wks	170	8	< 2	< 30	< 1	< 1	< 30	4	1	42,000	80	80
0	< 15	< 3	< 3	20	< 1	< 1	< 15	< 3	< 3	62,000	30	30
3 wks	780	< 2	< 2	20	< 3	< 3	< 15	< 3	< 3	120,000	60	50
4 wks	1,100	< 3	6	30	< 3	< 3	< 15	4	3	53,000	140	70
8 wks	< 30	7	2	< 30	1	2	< 30	3	8	41,000	50	130
12 wks	< 12	2	< 1	< 12	1	< 1	< 12	4	< 1	130,000	50	50
16 wks	120	8	< 1	20	4	< 1	< 12	7	< 1	70,000	90	20
24 wks	< 10	10	< 3	< 10	6	< 4	20	9	< 4	100,000	200	160
32 wks	350	4	< 1	< 10	3	< 1	< 10	5	2	170,000	150	270
36 wks	15	7	< 1	< 10	7	< 3	< 10	3	< 1	310,000	170	120
37 wks	< 10	5	< 1	3,600	9	< 4	< 10	2	< 1	460,000	160	110
39 wks	< 10	10	< 6	470	15	< 3	< 10	7	7	550,000	180	430
40 wks	20	4	< 1	320	7	< 4	< 10	2	< 1	650,000	180	290
41-43 wks^a^	< 10	7	< 2	20	15	< 1	< 10	6	< 1	530,000	280	470

Cell-free levels in plasma are expressed as RNA copies per ml plasma. Cell-associated levels are expressed as number of viral RNA and DNA copies per 100,000 cell equivalents. Values are given relative to the time of permanent tenofovir withdrawal (=time zero). Results of samples from other time points that are not shown in these tables were used for the analysis of Table 3.

^a^Values for 41–43 weeks after tenofovir withdrawal are those at the time of euthanasia.

Following tenofovir withdrawal on these 3 animals, viremia remained largely undetectable with transient blips. Animal 33088 fared the best, as viremia remained consistently below 50 copies per ml (Figure 1; Table 5); at 3 time points (at 1, 10 and 24 weeks of tenofovir withdrawal) plasma viremia was detectable but very low (20 to 30 copies per ml), while at the 37 other time points, viremia was below the detection limit (< 10–30 copies per ml; Figure 1, Table 5). Tissues collected at time of euthanasia had also very low or undetectable SIV RNA and DNA levels (Table 4).

The other 2 animals (30577 and 32186) had intermittent viral RNA blips > 50 copies/ml following tenofovir withdrawal. For these 2 animals the frequency of viral blips before and after withdrawal was similar (Table 3); however, after tenofovir withdrawal, the magnitude was higher, as both animals had RNA levels exceeding 1,000 copies/ml at one time point each. Animal 30577 had an initial detectable plasma viremia of 780 and 1,100 RNA copies per ml at 3 and 4 weeks after tenofovir withdrawal, respectively. Subsequently, viremia became undetectable for 11 consecutive weeks, followed by 2 transient blips (120 and 350 copies per ml at 16 and 32 weeks after withdrawal, respectively; Figure 1, Table 5). At the time of euthanasia (43 weeks after tenofovir withdrawal), animal 30577 had undetectable plasma viremia (< 10 copies/ml); while most tissues had detectable viral DNA, none had detectable viral RNA (Table 4).

Animal 32186 had several viral blips > 50 copies per ml after tenofovir withdrawal (Figure 1). An abrupt increase from undetectable (< 10 copies/ml at week 36 after withdrawal) to peak levels of 3,600 copies/ml (week 37 after withdrawal) was observed, after which virus levels decreased gradually to 20 copies/ml when the animal was euthanized at 41 weeks after tenofovir withdrawal (Figure 1, Table 5). Most tissues had low virus levels, with exception of the tonsil that had high SIV DNA and RNA levels (Table 4). Although the tonsil had normal histology and the animal did not have overt clinical signs, it is speculative that the animal may have had a pharyngeal infection, resulting in local immune activation and virus replication, approximately a month before euthanasia.

The last animal of the cohort, animal 33091, had a distinct outcome. After a 3-year period of undetectable plasma viremia (weeks 45–207 of SIV infection), this animal developed a gradual increase in viremia despite continued tenofovir treatment (Figure 1). When tenofovir was then withdrawn, instead of an abrupt viral rebound, viremia continued its gradual increase and was approximately 10-fold higher when the animal was euthanized 10 months later (Figure 1; Table 5). Virus could be detected readily in all lymphoid tissues, with the highest amounts in lymph nodes and tonsil (Table 4).

### Quantitation and genotypic analysis of K65R viral mutants

Real-time PCR methodology and population sequencing was used to monitor and quantitate K65R viral mutants after tenofovir withdrawal. For the animals with very low viremia, the plasma and tissue samples with the highest viral RNA were selected for this analysis. While plasma viral RNA levels in animals 30577, 33088 and 32186 were too low to amplify, viral RNA in the tonsil of animal 32186 revealed a pure K65R population with the additional RT mutations of N69S, I118V; these mutations were found previously in this animal [10]. For animal 33091, plasma collected at the time of tenofovir withdrawal and at the time of euthanasia (41 weeks after tenofovir withdrawal) had pure K65R populations; most other RT mutations were found in both samples (N69S, Y115F, I118V, D121H, V201A, S211N), while some mutations were found in only 1 sample (K40E and R82R/K at time of tenofovir withdrawal; K43E at time of euthanasia). Nearly all of these mutations have been described previously in K65R SIV isolates of animal 33091 and other tenofovir-treated animals [10,12,13,23]. In summary, there was no evidence of reversion from K65R to wild-type virus after tenofovir withdrawal.

### Frequency of lymphocyte populations and SIV-specific T cell responses correlate with levels of SIV replication

Throughout the time of tenofovir treatment and also after tenofovir withdrawal, the animals with suppressed viremia (29276, 30577, 32186 and 33088) maintained normal total lymphocyte counts, percentages and absolute values of CD4+ and CD8+ T lymphocytes, B lymphocytes and NK cells, and CD4+/CD8+ T cell ratios in peripheral blood (data not shown).

Animal 33091, consistent with its gradual increase in viremia, displayed a slowly progressive reduction in percentage and absolute CD4+ T lymphocytes and CD4/CD8 T cell ratio and an increase in the percentage of B lymphocytes in peripheral blood. At the time of tenofovir withdrawal, the animal had 9% CD4+ T lymphocytes (normal, mean +/− SD: 34-44%), 390 CD4+ T cells/μl (normal, mean−/+ SD: 325–785; a CD4/CD8 T cell ratio of 0.66 (normal range: 0.9-3.0); and 67% B lymphocytes (normal, mean −/+ SD: 25-38%). There was little change following tenofovir withdrawal and at the time of euthanasia, the corresponding values were 10.5% CD4+ T lymphocytes, 129 CD4+ T cells/μl; CD4+/CD8+ T cell ratio of 0.51; and 50% B lymphocytes. Percentages of CD8+ T lymphocytes and NK cells of animal 33091 were within the normal range, while absolute values were more variable (data not shown).

**Figure 2:**
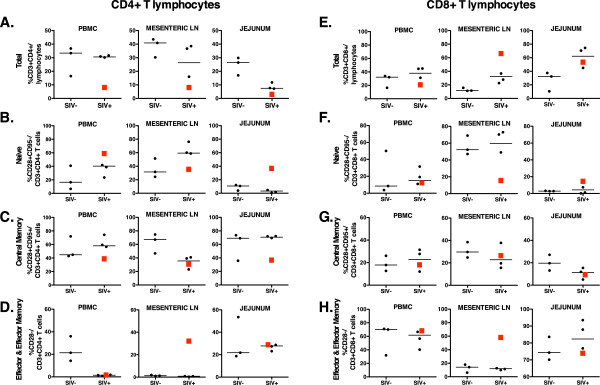
**T cell memory phenotyping of SIV-infected rhesus macaques after tenofovir withdrawal.** CD3 + 4+ T cells (**A**-**D**) and CD3 + 8+ T cells (**E**-**H**) from PBMC and tissue lymphoid cells collected at time of euthanasia were assessed via multiparameter flow cytometry. Values were compared to those of 3 uninfected animals. Data are reported as the frequency of CD4+ T cells (A) and CD8+ T cells (E) in the total lymphocyte population, or as the frequency of naïve cells, CD28 + CD95- (B,F), central memory cells, CD28 + CD95+ (C,G), and effector and effector memory cells, CD28- (D,H) within their respective CD4+ and CD8+ T cell populations. Animal 33091 is represented as a red square in all graphs.

Blood and tissues collected at time of euthanasia were available from 4 animals and used for more detailed lymphocyte phenotyping. As activated memory CD4+ T cells represent the main target cells of SIV, we examined the percentage of naïve T cells (CD28 + CD95-), central memory cells (CD28 + CD95+), and effector memory and effector cells (CD28-CD95+) (Figure 2). Compared to three uninfected age-matched adult macaques, and despite much variability among animals and tissues, the SIV-infected animals showed a trend towards lower CD4+ T cell populations in some tissues (such as total CD4+ T cells in jejunum; central memory CD4+ cells in the lymph nodes, and effector and effector memory CD4+ cells in peripheral blood), and a conversely higher percentage of CD8+ T cells and naïve CD4+ T cells in those tissues (Figure 2). Consistent with the moderate viremia, animal 33091 showed the lowest numbers of CD4+ T cells.

**Figure 3:**
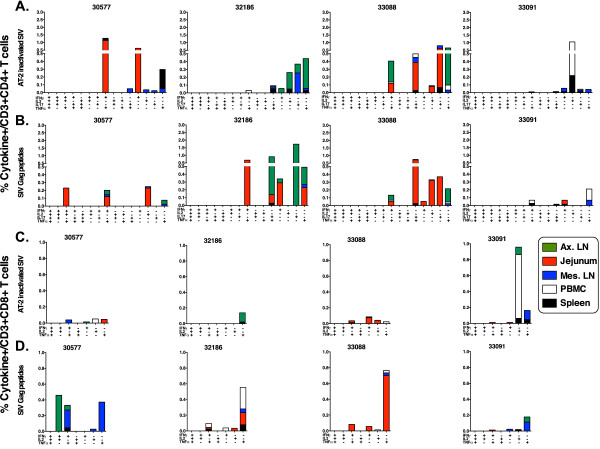
**SIV-specific T cell responses.** The response of CD3 + CD4+ (**A**, **B**) and CD3 + CD8+ (**C**, **D**) T cells after stimulation of PBMC or lymphoid cells with AT-2 (A,C) or SIVgag p27 overlapping peptides (B,D) and assessed for production of intracellular cytokines. Data are expressed as the frequency of cells positive for a set of cytokines, with tissue-specific responses represented by different colors: axillary lymph node (green), jejunum (red), mesenteric lymph node (blue), PBMC (white), spleen (black).

The frequency and quality of CD4+ and CD8+ SIV-specific responses were examined by measuring intracellular cytokines after *ex vivo* stimulation assay with SIVmac239 gag peptides and chemically-inactivated SIVmac239 (AT-2) (Figure 3). All SIV-infected animals had CD4+ and CD8+ SIV-specific responses to both stimuli, but the tissue distribution and range of functionality differed between animals. Viremic animal 33091 had less of a multifunctional response with more of the responding cells positive for only one cytokine compared to the other SIV-infected animals. Interestingly, animal 33091 lacked SIV-specific T cell responses in intestinal tissues compared to the other 3 SIV-infected animals that had better control of viremia (Figure 3).

**Figure 4:**
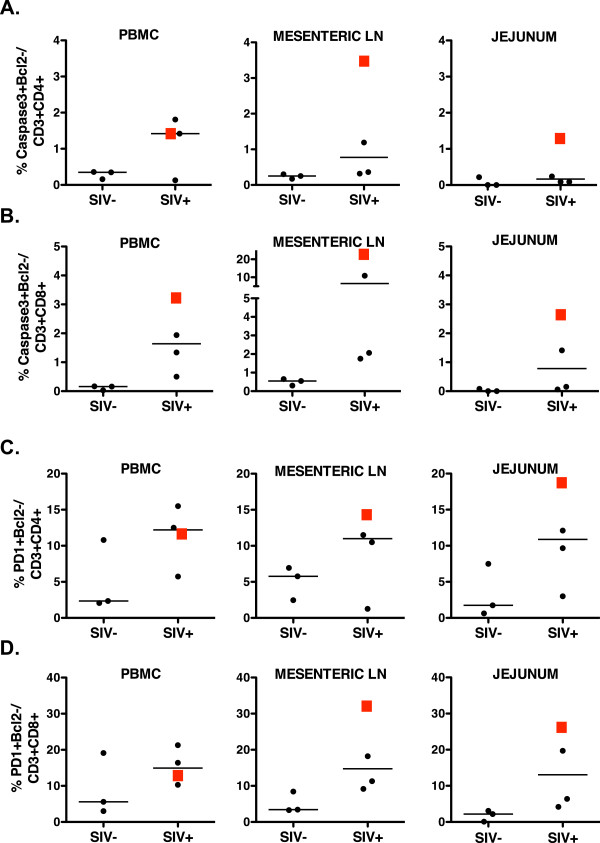
**T cell apoptosis and exhaustion phenotype in SIV-infected rhesus macaques after tenofovir withdrawal and uninfected macaques.** CD3 + 4+ T cells (**A**, **C**) and CD3 + 8+ T cells (**B**, **D**) from PBMC and tissue lymphoid cells were assessed via multiparameter flow cytometry for apoptotic cells, Caspase3 + Bcl2-, (A, B) and exhausted cells, PD-1 + Bcl2- (**C**, **D**). Data are reported as the frequency of the aforementioned cell populations in the CD4 + CD3+ or CD3 + CD8+ T cell populations, and are compared to those of 3 uninfected animals. Animal 33091 is represented as a red square in all graphs.

Loss of T cell function is often associated with T cell exhaustion. In fact, previous studies have demonstrated that chronic SIV infection is associated with the loss of the proliferation and the ability to produce cytokines, especially interleukin-2 [24,25], while the co-inhibitory molecule PD-1 is increased. Although all animals had a trend towards higher T cell exhaustion compared to uninfected animals, animal 33091 showed the highest level of T cell exhaustion, consistent with the quality of the SIV-specific T cell responses. Consequently, animal 33091 also had the highest frequencies of T cells undergoing apoptosis (Figure 4).

### Evaluation of SIV-specific antibody responses

Plasma of the 5 SIV-infected animals was tested by SIV-specific IgG ELISA for the detection of binding antibodies. All animals had measurable binding antibodies during tenofovir treatment (titers ≥ 409,600), with viremic animal 33091 having the highest titer (Table 6). For the 4 animals from which tenofovir was withdrawn, plasma of at least 12 time points after withdrawal was tested by ELISA, but SIV-binding antibody titers did not change significantly over time (Table 6).

**Table 6:** SIV-specific antibody responses in plasma at the end of tenofovir treatment and after tenofovir withdrawal

		**SIV-specific binding IgG**^1^	**Neutralizing antibody ID50 in TZM-bl cells**^**2**^				**ADCVI**^**3**^			
							**% inhibition of 1:100 serum dilution**			
**Animal number**	**Time point**		**SVA-MLV**	**SIVmac239CS.23**	**SIVmac251-CS**	**SIVmac251-TCLA**	**SIVmac251-CS**	**SIVmac251-TCLA**	**SIVmac251 (UCD)**	**RT-SHIV**
29276	Euthanasia	409,600	20	<10	17	29,462	48.89	99.98	99.99	nd
30577	TFV withdrawal	409,600	<10	<10	12	380,644	61.77	99.94	93.49	99.89
“	Euthanasia	409,600	<10	13	11	>1,562,500	33.29	99.94	94.48	99.89
32186	TFV withdrawal	1,638,400	<10	<10	10	>1,562,500	99.98	99.98	99.99	nd
“	Euthanasia	1,638,400	<10	<10	<10	>1,562,500	99.98	99.98	99.06	nd
33088	TFV withdrawal	409,600	<10	<10	13	451,911	99.98	99.98	99.99	nd
“	Euthanasia	409,600	<10	<10	<10	>1,562,500	99.78	99.98	99.99	nd
33091	TFV withdrawal	6,553,600	<10	<10	10	>1,562,500	99.98	99.98	99.99	nd
“	Euthanasia	6,553,600	<10	<10	12	>1,562,500	99.98	99.98	99.99	nd

^1^Values of SIV-binding antibody titers are the reciprocal of the highest plasma dilution (of 4-fold dilutions) that gave a positive signal on whole-virus antibody ELISA assay specific for IgG.

^2^Values of neutralizing antibody are the serum dilution at which relative luminescence units (RLUs) were reduced 50% compared to virus control wells (no test sample); viruses tested were pseudoviruses SIVmac239CS.23 and SVA-MLV grown in 293 T cells, and replication-competent SIVmac251-CS (hPBMC-grown) and uncloned SIVmac251-TCLA (H9-grown; tissue culture lab adapted).

^3^ADCVI: antibody-dependent cell-mediated virus inhibition was tested at a 1:100 serum dilution with SIV-infected CEM.NKr R5 as targets and human PBMC as effector cells. The reported values of % inhibition are the averages of 2 independent experiments. The SIVmac251CS and SIVmac251-TCLA are the same as those used in the neutralizing antibody assay; SIVmac251 (UCD) is a stock grown in rhesus PBMC derived from the same seed stock as the SIVmac251 stock that was used to inoculate animals 32186, 33088 and 33091. The RT-SHIV stock was grown in rhesus PBMC from the same seed stock used to infect animal 30577. Only serum of RT-SHIV infected animal 30577 was tested for ADCVI activity against RT-SHIV. nd indicates not done.

Serum samples were tested for neutralizing antibodies in a single-round infection assay that measures a reduction in luciferase reporter gene expression. Undetectable or very low titers of neutralizing antibodies were observed against env-pseudotyped viruses or human PBMC-grown SIVmac251. However, high titers of neutralizing antibodies were detected against laboratory adapted SIVmac251, and for animals 30577 and 33088, there was a more than 4-fold increase in titer from the time of tenofovir withdrawal to the time of euthania 43 weeks later (Table 6).

Serum samples were also tested for antibody-dependent cell-mediated virus inhibition (ADCVI), an antibody function that inhibits virus yield from infected cells in the presence of Fc-receptor bearing cells. All sera collected at the time of tenofovir withdrawal and at the time of euthanasia had very high ADCVI activity against all viruses tested, including viral stocks that were grown in human or rhesus PBMC (Table 6). There was no difference in ADCVI titers in sera of viremic animal 33091 in comparison to the other animals that controlled viremia.

### Histopathological evaluation at time of euthanasia

Viremic animal 33091, despite having a normal clinical appearance (normal weight, no overt evidence of opportunistic infections) had histological evidence of an intermediate stage of SIV disease progression, characterized by lymphoid hyperplasia and depletion and endocarditis (Table 7). In contrast, the 4 other SIV-infected animals with low or undetectable virus levels had no or mild lymphoid changes.

**Table 7:** Pathological observations at time of euthanasia

**Animal number**	**Most significant findings**
**29276**	Chronic interstitial nephritis with tubular atrophy
	Hypophosphatemic osteomalacia
	Mild generalized lymphoid hyperplasia
**30577**	Lymphofollicular hyperplasia, mild (inguinal, axillary, tracheobronchial, and mesenteric lymph nodes; MALT (mucosa-associated lymphoid tissue) of cecum and stomach) to moderate (tonsil, spleen, ileocecocolic lymph nodes).
**32186**	Kidney: polycystic chronic interstitial nephritis with tubular atrophy
	Lymph nodes: mild paracortical hyperplasia and mild loss of germinal centers
**33088**	Mild multifocal hepatitis, mild colitis, mild tracheitis
**33091**	Heart: valvular vegetative endocarditis
	Bone marrow and ileum: moderate multifocal lymphoid hyperplasia
	Lymph nodes: mild to moderate paracortical hyperplasia with indistinct germinal centers
	Spleen: mild lymphoid depletion

As described in detail previously [14,15], animals 29276 and 32186 had a long clinical history of renal toxicity caused by an initial prolonged high-dose tenofovir regimen, and potentially complicated by CD8+ cell depletion; despite relative stabilization after dosage reductions, it never resolved. Consistent with their clinical findings of glucosuria and elevated serum creatinine levels, the kidneys of both these animals showed chronic interstitial nephritis with tubular atrophy.

In contrast, the other 3 tenofovir-treated animals (30577, 33088 and 33091) had normal urinary and serum chemistry values, and had no gross or histopathological evidence of renal toxicity. This lack of detectable toxicity is reassuring considering that these animals were treated for many years with tenofovir regimens that gave exposures (as determined by plasma AUC values) higher than those of the clinical dose of tenofovir given to humans [15].

## Discussion

The current report is a follow-up study of 5 animals that received prolonged tenofovir monotherapy. Despite the emergence of K65R viral mutants with 5-fold reduced *in vitro* susceptibility, all five animals had eventually reached undetectable viremia [10,11,13,15]. Although we and others have previously described low or undetectable viremia after the emergence of K65R viral mutants in tenofovir-treated animals [12,26], the uniqueness of the present cohort of 5 animals resides in the unprecedented extensive period of tenofovir therapy (8 to 14 years) and survival. These animals were infected with virulent virus, and animals infected with these same viruses (wild-type or K65R) but not receiving any antiviral drug therapy were never able to spontaneously suppress viremia to low or undetectable levels and generally developed symptoms of AIDS within 2–24 months [10,11,13,15].

It is remarkable that during tenofovir treatment lasting 8 to 14 years, 4 of the 5 tenofovir-treated animals continued to have undetectable viremia with occasional viral blips. Similarly to observations in humans [27], the reasons for viral blips were not clear but may reflect a transient increase in viral production (e.g., due to immune activation) and/or minor fluctuations in antiviral effector mechanisms. Overall, the sustained suppression of virus replication in the 4 animals testifies to the strength of antiviral immune responses that were documented previously via CD8+ depletion experiments [10,11]. In other SIV studies, including many vaccine studies, animals that initially controlled replication of virulent virus often showed an increase in viremia after prolonged follow-up [28-31]. As explained in more detail elsewhere, it is plausible that, whereas as no single factor may be sufficient, a synergistic combination of (i) effective antiviral immune responses, preserved by early initiation of treatment, and sustained by ongoing low-level replication of K65R virus, (ii) a minor effect of K65R and compensatory mutations on viral replication fitness or diversity, and (iii) some residual drug efficacy against K65R mutants was responsible for a steady-state situation without viral rebound in these tenofovir-treated animals [9-11]. The demonstration that a combination of tenofovir and antiviral immune responses can suppress K65R SIV replication in macaques for many years is also consistent with the lack of viral rebound in treatment-experienced patients who develop K65R viral mutants during tenofovir treatment, and the observations that viremia in persons with detectable K65R mutants can be suppressed by tenofovir-containing regimens [32-34].

An exception was animal 33091, which despite having nearly 3 years of undetectable plasma viremia, slowly lost control of virus replication and demonstrated a slow disease progression while still on tenofovir treatment. It is unknown whether the transient CD8+ depletion that was performed previously in this animal at 2 weeks of SIV infection, when antiviral immune responses were in their early stages and probably most vulnerable, may have had a negative impact on the strength and breadth of the antiviral CD4+ or CD8+ cell repertoire, predisposing the eventual outgrowth of viral immune escape variants. In contrast, three of the four animals that demonstrated sustained suppression of viremia during tenofovir treatment had been depleted of CD8+ cells during a later stage of infection (≥ 39 weeks), when viremia was already suppressed by strong antiviral immune responses, and when the immune perturbation caused by CD8+ depletion may have had only a transient impact on antiviral immune responses.

For 3 of the 4 animals with sustained suppression of viremia during tenofovir treatment, the treatment was eventually stopped. Despite some transient periods of detectable low viremia, no rapid viral rebound was observed during the 10 months of observation. These animals that controlled viremia after tenofovir withdrawal also had low levels of viral DNA in blood and lymphoid tissues, stable CD4+ cell counts and a broad variety of cellular and humoral antiviral immune responses. These observations recapitulate the features of long-term non-progressor (LTNP’s), who naturally suppress virus replication for prolonged periods of time without antiretroviral drug intervention (reviewed in [35]).

The absence of a rapid rebound in all three animals can theoretically be due to low replication fitness of the virus, to strong antiviral immune responses, or to a combination of both. Previous experiments demonstrated that SIV and RT-SHIV isolates having the K65R mutation in combination with compensatory mutations have high replication fitness and virulence, and generally do not revert back to wild-type sequence following tenofovir withdrawal [10,11,13,16,17]. Also in the current study, withdrawal of tenofovir treatment did not lead to a detectable reversion from K65R to wild-type virus. Accordingly, the absence of a viral rebound likely represents an effective immune-mediated control of virus replication, rather than a major replication-attenuated phenotype. The development and maintenance of the cellular and humoral immune responses we observed must have been promoted by ongoing low-level replication of K65R viral mutants during the prolonged period of tenofovir treatment which created a balanced antigen expression/immune response steady-state.

Previously, depletion experiments revealed a major role for CD8+ cell-mediated immune responses in suppressing virus replication during tenofovir treatment in all 5 animals [10,11]. Because the cM-T807 antibody depletes both the CD8 + CD3+ T cells and the CD8 + CD3- NK cells, the relative contribution of these two cell subsets could not be established in those studies. As NK cells are also effector cells of ADCVI [36], they may play some role considering that the sera of all animals had high ADCVI activity, even against SIV strains that were poorly neutralized in the absence of effector cells. Even though animal 33091’s serum samples exhibited similarly high ADCVI activity *in vitro* as the other animals, it is unclear whether a reduced function of ADCVI effector cells *in vivo* contributed to its poorer control of virus replication. Additional CD8+ cell depletion experiments during the tenofovir-free period were not feasible because all five animals had developed antibodies against the cM-T808 antibody and other available CD8+ depleting antibodies (data not shown).

All SIV-infected animals had SIV-specific CD4+ and CD8+ cell-mediated responses. Although there was much variability in the functionality and tissue distribution among the different animals, the controlling animals had a trend towards a more multifunctional response that was located particularly in the intestinal tissues and lymph nodes, suggestive of an immunological control of virus replication. In contrast, animal 33091’s response was more mono-functional, absent in the intestinal tract, and residing predominantly in the peripheral blood, suggestive of an antigen-exposure driven immune response. These patterns are consistent with observations in other studies [37].

A closer look at the historical data of this cohort and other studies suggests several trends in the evolution of the immunological control of virus replication. Firstly, as shown in Figure 1, the viral rebound or blips shortly after tenofovir interruption in some animals in the current study were generally smaller than those observed with short-term treatment interruption (7 to 9 weeks) on these same animals earlier during the course of infection. In other animal studies, treatment interruptions also resulted in more rapid or higher viral rebound [10,38-54]. These observations suggest that the antiviral immune responses in these K65R virus-infected animals strengthened – rather than weakened- during the consecutive years of tenofovir treatment. Although this observation of strengthening immune responses provides hope, the time frame during which this was observed in the current study also highlights a research dilemma: future studies aimed at a functional cure, whether in animal models or human cohorts, may require a duration of more than 5 to 10 years, and therefore, a long-term investment in funding by research agencies.

Secondly, the optimism of the current results needs to be balanced with caution: although the frequency of low plasma RNA levels during the 10 months off tenofovir was similar to that during the prior period of tenofovir treatment, the magnitude of viremia was at times higher than what was observed during the preceding 5 years on tenofovir therapy. This is consistent with our previous observations that despite strong antiviral immune responses, there is still some benefit of continued tenofovir treatment, either by a residual direct antiviral effect and/or by immunomodulatory effects (reviewed in [9]). Although in the current study each transient increase of viral RNA was followed by a period of regained control, it is possible that during a much longer drug-free observation period such blips may become higher or more frequent and may require reinitiation of antiretroviral therapy to prevent disease progression.

It has to be re-emphasized that this cohort of animals had unique circumstances, namely prolonged tenofovir monotherapy, started relatively early in infection, in the presence of K65R viral mutants resulting in the generation and maintenance of effective antiviral immune responses and creation of a relative steady-state balance. Therefore, these results may not apply to HIV-infected patients who are started on ART late during infection, effectively control viremia and do not develop drug-resistant viral mutants. Such patients will typically show a rapid viral rebound upon drug withdrawal, possibly indicating insufficient antigenic exposure to generate, restore or maintain antiviral immune responses [55-58]. Consistent with our observation in monkeys, a minority of patients (5 out of 32) started on ART during primary HIV infection had good immunologic control of viremia after ART interruption, and these controllers had a trend toward earlier initiation and a longer duration of ART in comparison to noncontrollers or transient controllers [59].

The cohort of animals described here shares similarities with HIV-infected humans who, despite the presence of multi-drug resistant viral mutants, have low viremia associated with strong antiviral immune responses [60,61]. A difference, however, is that in the HIV-infected patients, interruption of nucleoside reverse transcriptase RT inhibitors led to an immediate and persistent increase in viremia and a reduction in CD4+ cell counts [62-64]. Potential reasons for this difference include (i) later initiation of ART, (ii) a much shorter duration of treatment, (iii) persistent detectable viremia (> 400–1000 copies/ml) while on ART (indicating less effective antiviral immune responses even prior to drug withdrawal), and (iv) regimens without tenofovir, which in addition to its antiviral activity has shown unique immunomodulatory properties in animal models [65,66].

For all these reasons, our results do not support withdrawing ART in HIV-infected individuals.

## Conclusions

By demonstrating a strong degree of viremia control associated with virus-specific immune responses following removal of antiviral drug treatment, the current study provides hope for an eventual functional cure, but warrants that much more research is required [4]. Animal models can continue to play a vital role in the development and screening of such strategies and to help advance concepts toward this functional cure.

## Methods

### Animals, virus inoculation, and tenofovir administration

All animals were rhesus macaques (*Macaca mulatta*) housed at the California National Primate Research Center (CNPRC), in accordance with American Association for Accreditation of Laboratory Animal Care Standards. We strictly adhered to the "Guide for the Care and Use of Laboratory Animals" prepared by the Committee on Care and Use of Laboratory Animals of the Institute of Laboratory Resources, National Resource Council [67].

Animals were inoculated intravenously or orally with virulent uncloned SIVmac251, the K65R-containing virus SIVmac385 (which is derived from SIVmac251), or RT-SHIV as described in their original studies [10,11,13]. Tenofovir was administered subcutaneously once daily, first at a regimen of 30 mg/kg, but doses were gradually reduced to a low maintenance regimen that gave plasma tenofovir concentrations and intracellular tenofovir diphosphate concentrations similar to or slightly higher than those observed in humans taking the oral prodrug tenofovir disoproxyl fumarate [15].

### Collection and processing of blood and tissue specimens

When necessary, animals were immobilized with ketamine HCL (Parke-Davis, Morris Plains, New Jersey) 10 mg/kg injected intramuscularly. Blood samples were collected regularly for monitoring viral and immunologic parameters as described previously [10]. Complete blood counts were performed on EDTA-anticoagulated blood samples. Samples were analyzed using an automated electronic cell counter (Baker 9000; Serono Baker Diagnostics) and from November 2002 onwards, a Pentra 60 C + analyzer (ABX Diagnostics); differential cell counts were determined manually.

Lymphoid tissues collected at euthanasia were processed to obtain cell suspensions by dissecting them with scalpels in RPMI 1640 (Invitrogen, Carlsbad, CA) supplemented with 10% fetal bovine serum (FBS; Gemini BioProducts, Calabasas, CA) (complete RPMI) and passing the cell homogenate through a cell strainer (Fisher, Pittsburgh, PA). Mononuclear cells were isolated from the splenic cell suspensions and the blood by density gradient centrifugation with Lymphocyte Separation Medium from MP Biomedicals (Aurora, OH), followed by two washes with RPMI 1640.

Cell isolation from intestinal tissues was performed according to previously described methods [68]. Briefly, ~2 in. pieces of the ileum and colon were rinsed with PBS and then minced using sterile scalpels. The tissue suspensions were placed in a shaking waterbath in RPMI 1640 containing 7.5% FBS and collagenase type II (0.5 mg/ml) for 30 minutes at 37^0^ C. After the digestion, the single cell suspension was passed through a 100 μm filter, spun down and resuspended in PBS. The remaining undigested tissue was resuspended in collagenase-media and the digestion step was repeated a total of 3 times. Mucosal lymphocytes were then isolated from the obtained single cell suspension by performing a 35%/60% Percoll (Sigma) gradient centrifugation. Intestinal lymphocytes were collected from the 35%/60% interface and washed twice with PBS before being resuspended in 10% FBS in RPMI 1640.

### Quantitation of viral RNA and DNA

Earlier data on plasma RNA levels in these animals were determined by quantitative branched-chain DNA (bDNA) assay for SIV, while the later plasma samples were tested by real-time reverse transcription-polymerase chain reaction (RT-PCR) assay for SIV *gag*, as described previously [10,11,69].

To determine cell-associated viral RNA and DNA levels, cell pellets of approximately 2 million PBMC or lymphoid cells isolated from lymphoid tissues at time of euthanasia were snapfrozen and stored at -70 °C. The cell pellets were subsequently tested for SIV *gag* RNA and DNA and CCR5 DNA (as a reference for cell equivalents, because of 2 copies of CCR5 per cell) according to methods described previously [70].

### Real-time PCR for sensitive detection of K65R viral mutants

The real-time PCR methodology to quantitate K65R mutants of SIV and RT-SHIV has been described previously [11,71]*.*

### MHC typing

MHC typing for 9 class I alleles (Mamu-A*01, A*02, A*08, A*11, B*01, B*03, B*04, B*08, B*17) was performed using methods previously described [19,72].

### Phenotyping of lymphocyte populations

Multiparameter flow cytometric analysis was performed to characterize lymphocyte populations in PBMC and tissue cell suspensions. All antibodies were from BD Biosciences (San Jose, CA) unless otherwise stated. To define T and B lymphocytes and NK cells, 4-color flow cytometry techniques, consisting of a single tube containing antibodies to CD3, CD4, CD8 and CD20, were used and samples were analyzed on a FACSCalibur flow cytometer, as described previously [10]. CD4+ T lymphocytes, CD8+ T lymphocytes, NK cells and B cells were defined as CD4 + CD3+, CD8 + CD3+, CD8 + CD3- and CD20 + CD3- lymphocytes, respectively.

In different tubes, T cell sub-populations of CD3+ (clone SP34-2) and CD4+ (clone L200) or CD8+ (clone SK1) T cells were further defined by markers of memory [CD28 (clone CD28.2) and CD95 (clone DX2)] and apoptosis [PD1 (clone J105, eBioscience), Bcl-2 (clone Bcl-2/100), and Caspase 3 (clone C92-605)]; samples were acquired on a FACS ARIA (BD), and data were analyzed using FlowJo software (TreeStar, Ashland, OR). Data are reported as the frequency of lymphocytes that stained positive for a defined set of markers.

### Antigen-specific stimulation of lymphocytes

Intracellular cytokine production was assessed via multiparameter flow cytometry from fresh PBMC and lymphoid cells after stimulation with SIV antigens as reported previously [73]. Briefly, 1x10^6^ cells per tube were stimulated with 300 ng/mL aldrithiol-2 (AT-2)-inactivated whole SIVmac239 (provided by Dr. J. Lifson, NCI) or with 5 μg/mL of a pool of overlapping 15-mer peptides spanning the SIVgag p27 protein (provided by the NIH Reference and Reagent Program). PMA (50 ng/mL, Sigma) and ionomycin (1 μg/mL, Sigma) were used as a positive control. Cells were stimulated in the presence of anti-CD49d, anti-CD28 (0.5 μg/mL each) and monensin (2 μM; eBioscience). Cells were incubated at 37 °C in 5% CO_2_ for 6 hours, and after 1 hour, Brefeldin A (10 μg/mL) was added. After stimulation, cells were stained with the following antibodies following BD staining protocols: CD3 (clone SP34-2), CD4 (clone L200), IL2 (clone MQ1-17 H12), TNF-α (clone MAB11), IFN-γ (clone B27), and IL17a (clone eBio64CAP17, eBioscience). Samples were acquired on a FACS ARIA (BD), and data were analyzed using FlowJo software (TreeStar, Ashland, OR). Data are reported as the frequency of CD3 + CD4+ or CD3 + CD4- T cells that stained positive for a defined combination of intracellular cytokines. Frequency values were considered positive if they were 2-fold greater than that of medium-only cultures and if the subtracted value of stimulation cultures minus media-only cultures was greater or equal to 0.01%.

### Detection of SIV-specific antibody responses

SIV-specific immunoglobulin G (IgG) in plasma samples was detected by ELISA as decribed previously [23]. Briefly, Costar EIA/RIA plates (Fisher Scientific, Santa Clara, CA) were coated with purified whole SIVmac251, and serial 4-fold dilutions of plasma or serum were tested. Titers were determined as the highest dilution with an OD above cut-off value.

### Neutralization assay

Neutralization was measured as a reduction in luciferase reporter gene expression after a single round of infection in TZM-bl cells as described [74,75]. TZM-bl cells were obtained from the NIH AIDS Research and Reference Reagent Program, as contributed by John Kappes and Xiaoyun Wu. Briefly, 200 TCID_50_ of virus was incubated with serial 3-fold dilutions of test sample in duplicate in a total volume of 150 μl for 1 hr at 37 °C in 96-well flat-bottom culture plates. Freshly trypsinized cells (10,000 cells in 100 μl of growth medium containing 75 μg/ml DEAE dextran) were added to each well. One set of control wells received cells + virus (virus control) and another set received cells only (background control). After a 48 hour incubation, 100 μl of cells was transferred to a 96-well black solid plates (Costar) for measurements of luminescence using the Britelite Luminescence Reporter Gene Assay System (PerkinElmer Life Sciences). Neutralization titers are the dilution at which relative luminescence units (RLU) were reduced by 50% compared to virus control wells after subtraction of background RLUs. Assay stocks of molecularly cloned Env-pseudotyped viruses were prepared by transfection in 293 T cells and were titrated in TZM-bl cells as described [75]. An assay stock of uncloned SIVmac251 was produced in H9 cells and titrated in TZM-bl cells.

### Antibody-dependent cell-mediated virus inhibition

Antibody-dependent cell-mediated virus inhibition (ADCVI) was measured using methods similar to those described previously [36]. Briefly, target cells were prepared by infecting CEM.NKr.CCR5 cells (AIDS Reagent Program) with SHIV or SIV for 72 hours. After washing, target cells were mixed with fresh human PBMC effector cells at an effector:target ratio of 10:1 in the presence of test sera at a final concentration of 1:100. Wells were washed at day 3 to remove antibody and fresh medium was then added. Supernatant fluid was assayed at day 7 for p27 by ELISA (Zeptometrix). Percent inhibition (compared to negative control) was calculated as previously described [36].

### Histopathological evaluation

At time of necropsy, an evaluation of gross pathology was performed. In addition, tissues were collected in 10% buffered formalin, embedded in paraffin, sectioned, stained with hematoxylin-eosin, and examined by light microscopy.

### Statistical analysis

Statistical analyses were performed with Prism 5 for Mac and Instat 3 (GraphPad Software Inc., San Diego, CA). A value of *P* of < 0.05 was considered statistically significant.

## Competing interests

This study was partially supported by Gilead Sciences. KVR holds stocks of Gilead Sciences.

## Author’s contributions

KVR was responsible for the overall design of the study, coordination and data analysis, and drafted the manuscript. KT and KA participated in the design, performed and analyzed the flow cytometry-based immune assays. KJ and YG participated in sample processing and data analysis. CL and DM performed the neutralizing antibody assays. JJ, JL and WH performed the K65R analysis. GL and DF performed the ADCVI assays. RP and DC performed all gross- and histopathology. All authors read and approved the final manuscript.
